# Enzyme-dependent fluorescence recovery of NADH after photobleaching to assess dehydrogenase activity of isolated perfused hearts

**DOI:** 10.1038/srep45744

**Published:** 2017-03-31

**Authors:** Angel Moreno, Sarah Kuzmiak-Glancy, Rafael Jaimes, Matthew W. Kay

**Affiliations:** 1 Department of Biomedical Engineering, The George Washington University, Washington, DC 20052, USA.

## Abstract

Reduction of NAD^+^ by dehydrogenase enzymes to form NADH is a key component of cellular metabolism. In cellular preparations and isolated mitochondria suspensions, enzyme-dependent fluorescence recovery after photobleaching (ED-FRAP) of NADH has been shown to be an effective approach for measuring the rate of NADH production to assess dehydrogenase enzyme activity. Our objective was to demonstrate how dehydrogenase activity could be assessed within the myocardium of perfused hearts using NADH ED-FRAP. This was accomplished using a combination of high intensity UV pulses to photobleach epicardial NADH. Replenishment of epicardial NADH fluorescence was then imaged using low intensity UV illumination. NADH ED-FRAP parameters were optimized to deliver 23.8 mJ of photobleaching light energy at a pulse width of 6 msec and a duty cycle of 50%. These parameters provided repeatable measurements of NADH production rate during multiple metabolic perturbations, including changes in perfusate temperature, electromechanical uncoupling, and acute ischemia/reperfusion injury. NADH production rate was significantly higher in every perturbation where the energy demand was either higher or uncompromised. We also found that NADH production rate remained significantly impaired after 10 min of reperfusion after global ischemia. Overall, our results indicate that myocardial NADH ED-FRAP is a useful optical non-destructive approach for assessing dehydrogenase activity.

Fluorescence recovery after photobleaching (FRAP) is a popular approach for studying the temporal dynamics and diffusion kinetics of fluorescent and fluorescently labeled molecules within living cells^1^. Enzyme-dependent FRAP (ED-FRAP) is useful for measuring enzyme kinetics and involves the photolysis of a product of an enzyme reaction and the subsequent analysis of the replenishment of that product^2^,^3^,^4^. ED-FRAP is particularly useful for studying the rate of production of NADH, an important reducing agent and endogenous fluorophore (excitation 360 nm, emission 460 nm). NAD^+^ is reduced by cellular dehydrogenase enzymes to generate NADH and the NAD^+^: NADH redox couple is a highly regulated component of cellular metabolism^5^,^6^,^7^. NADH drives ATP production in the mitochondria and the NAD^+^: NADH ratio reflects the balance between the reduction of NAD^+^ via substrate oxidation and the oxidation of NADH for oxidative phosphorylation^8^.

In cardiomyocytes, the NAD^+^: NADH ratio is dependent upon multiple factors^9^, including available substrate^8^,^10^,^11^,^12^, cardiac work^5^,^13^, and myocardial oxygenation^13^,^14^. NAD^+^: NADH ratios reported in the literature for normoxic perfused hearts range from 22% reduced^15^ to as high as 80% reduced^10^. Cellular NAD^+^ and NADH concentrations can be measured using absorbance assays but this typically requires cellular manipulation that destroys cells^16^, eliminating the ability to measure concentration changes over time from living cells. A non-destructive alternative is to measure NADH fluorescence (fNADH) from living cells and tissue, which provides a quantitative assessment of the NAD^+^: NADH ratio that is consistent with that provided by biochemical assays of NAD^+^ and NADH concentrations^17^.

NADH fluorescence is typically monitored from cardiac tissue by illuminating tissue with low intensity ultraviolet (UV) light and acquiring the resulting fluorescence between 450–475 nm^5^,^8^,^11^,^13^,^14^,^18^. Our recent studies in perfused hearts demonstrate how epicardial fNADH fluctuations reveal changes in NADH production and consumption (oxidation) during physiologic perturbations^11^,^13^. An important limitation is that such fluctuations are the combination of NADH production and NADH consumption, so neither the rate of NADH production, nor the rate of consumption, can be independently measured using fluorescence alone. For example, an increase in fNADH could indicate either an increase in the rate of NADH production, a decrease in the rate of NADH consumption, or be an indication of an oxygen limitation, resulting in progressive NADH production with negligible consumption until reaching the fully reduced state.

Using cellular preparations and isolated mitochondria suspensions, Combs and Balaban measured the rate of fNADH recovery after NADH photolysis to introduce NADH ED-FRAP as an assessment of dehydrogenase activity^2^,^3^. They showed that the rate of fNADH recovery, measured within the setting of steady-state NADH consumption, was proportional to the activity (concentration) of glutamate dehydrogenase (GDH), a mitochondrial enzyme that produces NADH during amino acid catabolism. The advantages of NADH ED-FRAP are that NAD^+^ is produced by the photolysis of NADH without destroying NADH molecules^3^; the kinetic properties of the tricarboxylic acid (TCA) cycle are not altered^2^; and most of the fNADH signal is confined to the mitochondria^19^ because NADH does not diffuse from adjacent regions, in contrast to standard FRAP techniques^20^,^21^. As a result, fNADH recovery during NADH ED-FRAP is dominated by the production of NADH by the dehydrogenase enzymes of the TCA cycle. With these advantages, NADH ED-FRAP provides additional insight into myocardial energetics, above that of monitoring unbleached fNADH, by providing a direct assessment of the rate of mitochondrial NADH production.

However, to date, no studies have demonstrated how the rate of NADH production could be assessed in perfused hearts using NADH ED-FRAP^22^. This would be an important advancement because perfused hearts are a popular experimental approach for studying electromechanical function, metabolism, and arrhythmias, primarily because precise control of experimental variables such as fuel supply, pressure, and temperature can be maintained^11^,^13^,^23^,^24^. Therefore, the goal of the present study was to expand the technique of NADH ED-FRAP to perfused hearts and use it to study the rate of NADH production during controlled metabolic perturbations.

We demonstrate for the first time that NADH ED-FRAP is a useful approach for measuring the rate of NADH production in contracting perfused hearts. The approach was implemented using high-power UV light-emitting diodes (LEDs) and a high-speed CCD camera. The optimal energy delivery of 23.8 mJ of UV light (367.5 ± 5.5 nm) was found by modulating the light intensity and pulse width while measuring the NADH photobleaching fraction and ensuring adequate recovery of fluorescence under controlled conditions. We also tested the efficacy of NADH ED-FRAP for measuring changes in NADH production rate in hearts perfused at low temperatures, in the presence and absence of mechanical contraction, and before and after ischemia/reperfusion injury. These new results provide insight into how NADH production rate is adjusted to couple with the energy demand of specific physiologic conditions.

## Results

**Figure 1:**
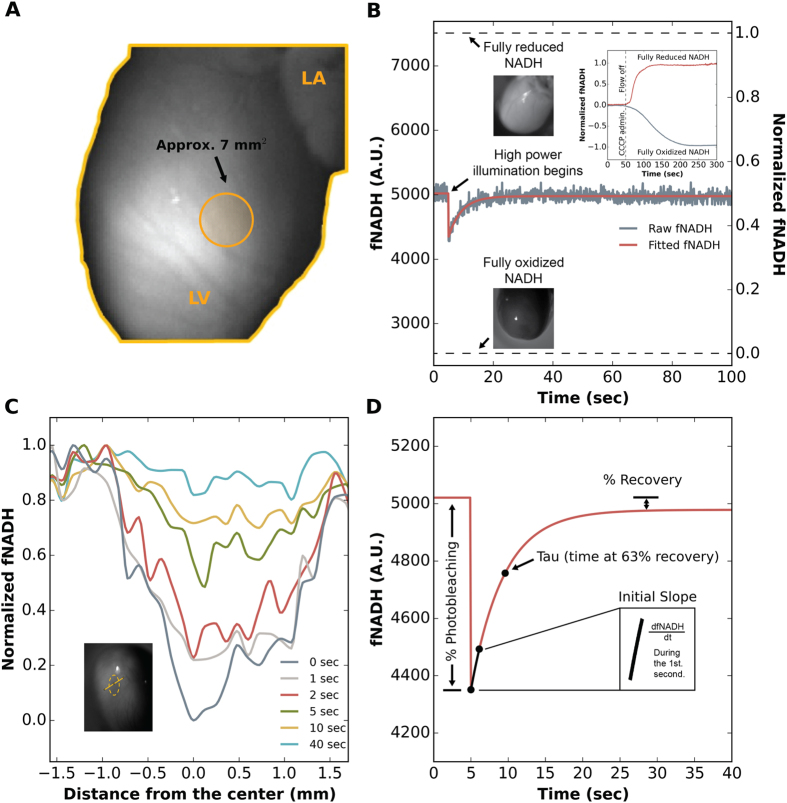
(**A**) Photobleaching UV light was focused on the LV epicardium within an area of ~7 mm^2^ (orange circle) and delivered as one of four modes (Table 1). (**B**) A typical NADH ED-FRAP signal is plotted within the range of the estimated total NADH pool. The application of 23.8 mJ of photobleaching energy typically lowered baseline fNADH by 13.2 ± 2.3%. **Inset**: Normalized fNADH signals are shown for one heart during full reduction (global ischemia at t = 50 sec) and for another heart during full oxidation (CCCP administered at t = 50 sec). The non-normalized signals were used to estimate the total NADH pool. (**C**) An example of spatial fNADH recovery profiles from 0 to 40 sec after photobleaching the area shown in the fNADH image (yellow dotted line). Within 40 sec the spatial fNADH profile returned to baseline levels and was indistinguishable from the fNADH of surrounding unbleached tissue. (**D**) Measurements extracted from NADH ED-FRAP signals: (1) Percent photobleaching was measured from baseline (fNADH at t < 5 sec) to the maximum fNADH drop (fNADH at t = 5 sec); (2) Percent recovery was measured from baseline to the steady-state fNADH recovery value; (3) Recovery time constant tau was measured as the time to 63% of full fNADH recovery; (4) The initial slope of recovery was measured as the straight-line slope of change in fNADH during the first second of recovery.

Our NADH ED-FRAP approach for measuring dehydrogenase activity in isolated perfused hearts is presented in Fig. 1. High power UV light was focused on the LV epicardium to photobleach NADH within an area of ~7 mm^2^ (Fig. 1A). The LV epicardium was then illuminated with low power UV light to image fNADH (Fig. 1B). Recovery kinetics of fNADH were measured from the images within a region of interest selected to contain the photobleached tissue (Fig. 1C,D).

### Estimated percentage of NADH pool photobleached

Studies were conducted (n = 8) to measure the percentage of the total NADH pool ([NAD^+^] + [NADH]) that was photobleached during a typical NADH ED-FRAP measurement. This was done by imaging epicardial NADH fluorescence after full NADH oxidation (via administering CCCP) and after full NADH reduction (during global ischemia) to assess the full range of fNADH. During control conditions, average baseline fNADH before photobleaching indicated that the NAD^+^: NADH redox poise of epicardial tissue in our studies was 50.7 ± 4.5% reduced and 49.3 ± 4.5% oxidized (Fig. 1B). These values are consistent with previous studies in isolated hearts where levels of fNADH indicated the redox poise to be within the range of 50% reduction when glucose was the only exogeneous substrate^10^. This is also consistent with whole-cell NAD^+^: NADH ratios measured using an absorbance assay kit for myocytes isolated from healthy mouse hearts^16^. The ratio was approximately one, indicating a redox poise for isolated cardiomyocytes to be 50% reduced and 50% oxidized. In our studies, we found that the application of 23.8 mJ of photobleaching energy lowered baseline fNADH by 13.2 ± 2.3%, thereby increasing the redox poise of the photobleached tissue to 62.5 ± 3.8% oxidized (Fig. 1B).

### Total energy delivered (TED) during NADH ED-FRAP

**Figure 2:**
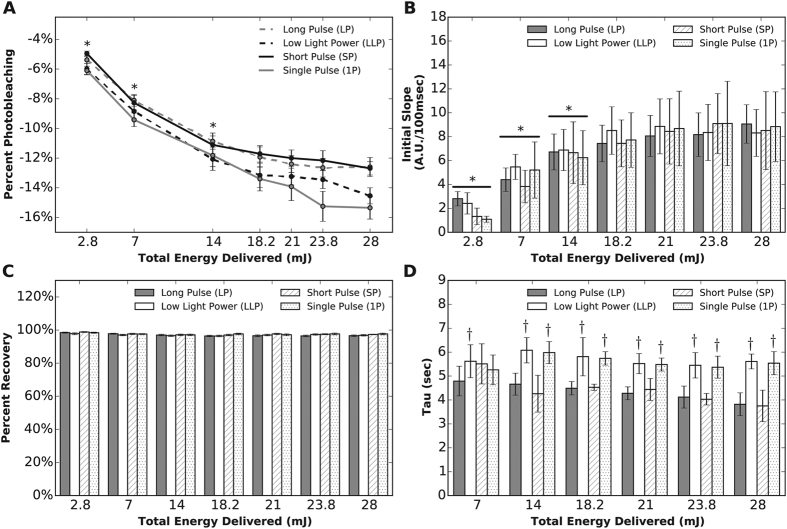
The effect of photobleaching mode (Table 1) and total energy delivered (TED) on NADH ED-FRAP measurements during control conditions in perfused hearts (n = 5 for each mode at each TED). (**A**) Percent photobleaching is plotted for each TED between 2.8 and 28 mJ for the four photobleaching modes. For each mode, photobleaching increased until 18.2 mJ, where it reached a maximum. Astericks indicate significant differences within each mode for comparisons between each TED and a TED of 23.8 mJ. (**B**) Initial slope of fNADH recovery plotted for TED between 2.8 and 28 mJ for the four photobleaching modes. Initial slope increased within each mode until 18.2 mJ, where it reached a maximum. Astericks indicate significant differences within each mode for comparisons between each TED and a TED of 23.8 mJ. (**C**) Percent recovery plotted for TED between 2.8 and 28 mJ for the four photobleaching modes. For all modes, percent recovery approached 100% and was not dependent upon TED (p > 0.05). (**D**) Recovery time constant tau plotted for TED between 2.8 and 28 mJ for the four photobleaching modes. Tau varied with photobleaching mode for a particular TED but the effect of TED on tau within each photobleaching mode did not reach statistical significance (p > 0.05). Crosses indicate significant differences for each TED for tau measured using LLP, SP, and 1 P photobleaching modes compared to tau measured using the LP mode.

We found that a minimum TED must be administered to the epicardial surface to achieve repeatable NADH photobleaching. Increasing TED from 2.8 to 18.2 mJ increased percent photobleaching for each photobleaching mode (Long Pulse - LP, Short Pulse - SP, Low Light Power - LLP and Single Pulse - 1P) (p < 0.001) (Fig. 2A). As TED was increased from 18.2 to 28 mJ, there was little increase in percent photobleaching. For all modes, percent photobleaching for TED greater than 18.2 mJ was compared to percent photobleaching at a TED of 23.8 mJ and the differences were not significant (p > 0.05), indicating that maximal photobleaching could be assured with a TED of 23.8 mJ. The exception was when TED was delivered with the 1P mode: an increase in TED above 18.2mJ increased percent photobleaching beyond that of the SP and LP modes (p < 0.05) (Fig. 2A). However, for the 1P mode, percent photobleaching for TED equal to or greater than 18.2 mJ was not significantly greater than that of 23.8 mJ.

The initial slope of recovery was also dependent upon the TED. Initial slope increased as TED was increased from 2.8 to 14 mJ across all photobleaching modes, with initial slope remaining constant as TED was increased from 18.2 to 28 mJ (Fig. 2B). Unlike percent photobleaching and initial slope, the percent recovery of fNADH was very consistent for all TEDs (Fig. 2C). When grouping all photobleaching modes together, percent recovery was 98.4 ± 0.45% at the lowest TED, and only decreased slightly to 97.2 ± 0.31% at the highest TED.

Significant variability was observed across photobleaching modes in measurements of the recovery time constant tau (Fig. 2D). For all modes at a TED greater than 7 mJ, tau was consistently shorter when the TED was delivered with the LP or SP modes compared to LLP and 1 P modes (Fig. 2D) (p < 0.001). Tau for LLP and 1P modes was significantly longer than that of LP (p < 0.001), meaning fNADH recovery was slower for these photobleaching modes. These differences occurred despite equal initial slopes of recovery for all photobleaching modes at the same TED (Fig. 2B). Comparing tau within individual photobleaching modes across all TEDs did not indicate that TED had a significant effect on tau.

### NADH ED-FRAP and tissue viability

**Figure 3:**
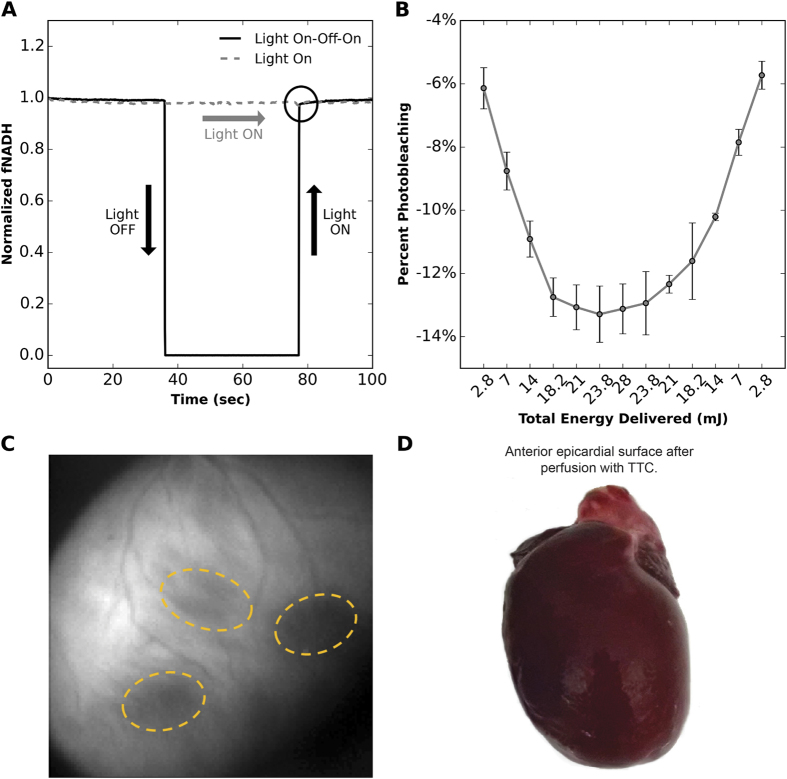
Sustained NADH photolysis and tissue viability after NADH ED-FRAP. (**A**) Normalized fNADH is plotted to illustrate that low power UV illumination (1.5 mW) used to image fNADH does not cause measurable NADH photobleaching in excised hearts. A sequence of light on-off-on shows that fNADH after continuous illumination matches that of fNADH immediately after a 40 sec interval of darkness (circled). (**B**) Percent photobleaching is not altered by previous applications of photobleaching light (LP mode). Results are shown for a “round trip” experiment where TED was increased from 2.8 to 28 mJ then decreased from 28 to 2.3 mJ for the same region of tissue (n = 3 hearts). (**C**) Sustained NADH photolysis was observed (indicated by a sustained drop in fNADH) after applying six rounds of high energy (18.2–28 mJ, LP mode) to the same areas of tissue (circled). (**D**) Less than six applications of high energy (18.2–28 mJ, LP mode) to the same region of tissue did not affect tissue viability, as evidenced in the image shown by the absence of pale epicardial tissue within photobleached regions after TTC staining.

Studies were conducted to verify that the low power UV illumination (1.5 mW) used to image epicardial fNADH would not cause tissue photodamage or significant NADH photobleaching. First, fNADH was continuously acquired from an epicardial region of interest while illuminating the epicardium for 35 sec, then the low power UV light was turned off for 40 sec and back on for another 35 sec. fNADH was acquired again from the same region of interest during 100 sec of constant illumination, which is the time typically required for one NADH ED-FRAP measurement. fNADH signals from a study are shown in Fig. 3A, which indicate that NADH fluorescence is not lower when the low power UV light was on compared to what it would be if the light were off (circled region in Fig. 3A). Similar studies were completed by Combs and Balaban in isolated myocytes and demonstrated a slow constant decline of fNADH during steady-state UV illumination, mainly due to the balance between the net product of NADH photolysis and its metabolic production^2^. In contrast, our measurements in perfused hearts indicate stable fNADH during steady-state illumination without detectable photobleaching caused by low power UV illumination.

Epicardial tissue remained viable after several rounds of photobleaching, even after the maximal TED of 28 mJ was applied. Percent photobleaching, tau, initial slope of recovery, and percent recovery in a photobleached region were not altered by previous NADH ED-FRAP applications. This is shown for percent photobleaching in Fig. 3B, where TED for a single site was increased from 2.8 to 28 mJ then decreased from 28 to 2.8 mJ. This “round-trip” NADH ED-FRAP shows that photobleaching percentage is dependent upon the amount of energy imparted to the heart but not the order in which it is applied. However, we did observe that repeated application of high power UV light to the same area caused irreversible NADH photolysis. This is evident after 6–8 rounds of ED-FRAP at high energies (18.2–28 mJ), as shown in Fig. 3C. Four applications of such high energy caused a sustained fNADH loss of up to 10% with respect to the first application. Even so, triphenyltetrazolium chloride (TTC) staining indicated that the tissue remained viable (Fig. 3D). Altogether, these results indicate that NADH ED-FRAP at TED between 18.2–28 mJ should be limited to no more than three or four applications per site to avoid sustained NADH photolysis.

**Figure 4:**
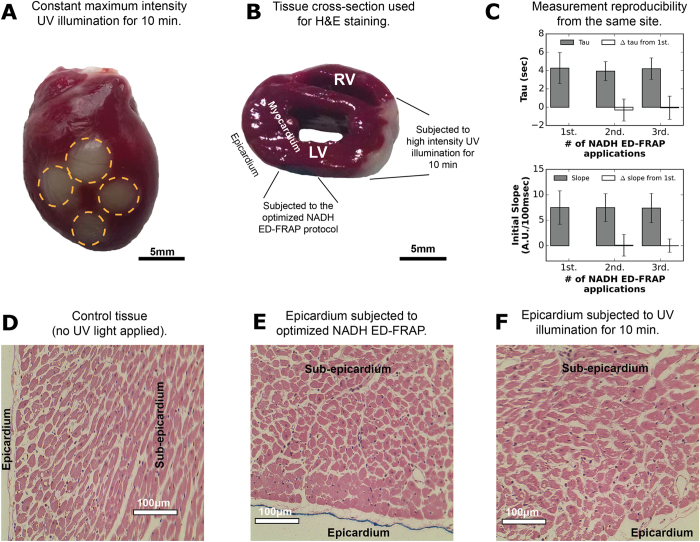
Assessment of UV illumination effects on tissue viability and cell morphology. **(A)** After TTC staining of positive controls, non-viable epicardial tissue was revealed at sites exposed to 10 min of continuous UV illumination at max power (500 mW). **(B)** Cross-section of a heart after TTC staining reveals the transmurality of non-viable tissue in positive controls exposed to high intensity UV illumination for 10 min (500 mW). The high intensity illumination damaged the tissue up to a depth of approximately 0.5 mm. Adjacent tissue exposed to the optimized NADH ED-FRAP protocol (LP mode) remained viable with no visible evidence of epicardial or transmural damage. **(C)** No difference in measurements of NADH production rate were detected (p > 0.05) after three applications of NADH ED-FRAP to the same epicardial site. Five minutes elapsed between each measurement. Tau (top) and initial slope of recovery (bottom) were the same after the second and third NADH ED-FRAP measurement compared to the first measurement. Error bars in this panel correspond to standard deviation. **(D)** H&E staining of an LV short axis section within the area that was not illuminated. As expected, no changes in cellular morphology were observed. **(E)** H&E staining of an LV short axis section within the area that was exposed to three applications of NADH ED-FRAP using the LP photobleaching mode. No changes in cellular morphology were observed. **(F)** H&E staining of an LV short axis section within the area that was exposed to high intensity UV illumination for 10 min (500 mW). No changes in cellular morphology were observed even though TTC staining revealed that this tissue was not viable, as shown in Panel B.

Epicardial tissue exposed to the LP photobleaching mode did not exhibit evidence of cellular damage in either TTC staining assessments (Figs 3D and 4B) or haematoxylin and eosin (H&E) cellular histology assessments (Fig. 4E). NADH ED-FRAP was repeated three times at same epicardial site to further confirm that the LP photobleaching mode did not detrimentally impact tissue viability and measurement repeatability. Tau and initial slope of recovery were compared after each application (Fig. 4C). No significant difference (p > 0.05) was detected when measurements from the second and third application were compared to the first application. This suggests that tissue viability was not detrimentally altered after three or less applications of NADH ED-FRAP. As a positive control, other hearts were subjected to 10 min of continuous UV illumination at the maximum LED power (500 mW). TTC and H&E assessments were repeated. As expected, TTC staining revealed metabolically inactive tissue within the areas that were subjected to the 10 min of 500 mW UV illumination (Fig. 4A,B). However, histological analysis did not reveal cellular morphology changes and many nuclei maintained a highly defined and normal shape (Fig. 4F).

### Dehydrogenase activity and temperature

**Figure 5:**
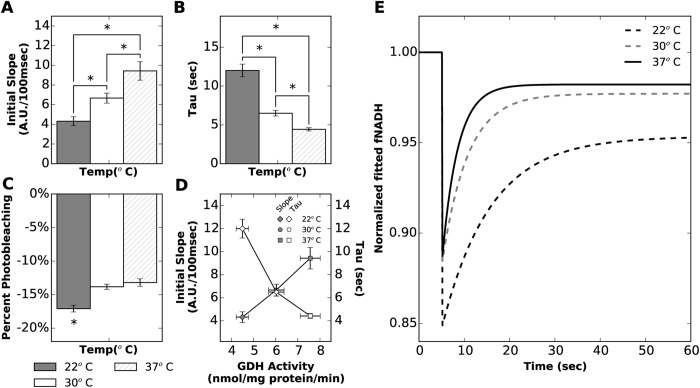
NADH ED-FRAP measurements (LP mode) for hearts perfused at three temperatures. Hearts were electromechanically uncoupled with BDM. (**A**) The initial slope of fNADH recovery increases with temperature (p < 0.001, n = 5). (**B**) The time constant of fNADH recovery (tau) drops with increasing temperature (p < 0.001, n = 5) (**C**) Percent photobleaching is greater at 22 °C (p < 0.001, n = 5) but not significantly different at 30 & 37 °C. (**D**) Initial slope and tau measured from hearts perfused at 22, 30, and 37 °C are plotted with GDH activity (n = 4) measured at the same temperatures. (**E**) Representative fNADH data acquired during NADH ED-FRAP were fitted (‘y’ in Equation 4) and plotted for the three temperatures studied.

Hearts were perfused at three temperatures to evaluate the effect of temperature on NADH production rate. The rate of fNADH recovery after photobleaching increased as temperature increased from 22 to 37 °C (Fig. 5A,B). Although percent photobleaching was not different at 30 & 37 °C, photobleaching was greater at 22 °C compared to higher temperatures (Fig. 5C) (p < 0.001). This is likely the result of slowed NADH production at lower temperature. Photobleaching during slow NADH production would achieve a higher level of NADH photolysis, thereby increasing percent photobleaching. Of note is that fNADH only recovered to 95.12 ± 0.45% at 22 °C compared to 97.11 ± 0.34% and 96.58 ± 0.42% at 30 & 37 °C, respectively, likely due to slow NADH production at low temperatures. Glutamate dehydrogenase (GDH) activity was measured in enriched mitochondrial fractions at the same three temperatures to validate the NADH ED-FRAP results. GDH activity significantly increased from 4.50 ± 0.28nmol/mg-protein/min at 22 °C to 6.03 ± 0.35 and 7.56 ± 0.45 nmol/mg-protein/min at 30 & 37 °C (p < 0.001), respectively. These values were highly correlated with values of fNADH initial slope and tau measured via NADH ED-FRAP (Fig. 5D).

### Dehydrogenase activity and contraction

**Figure 6:**
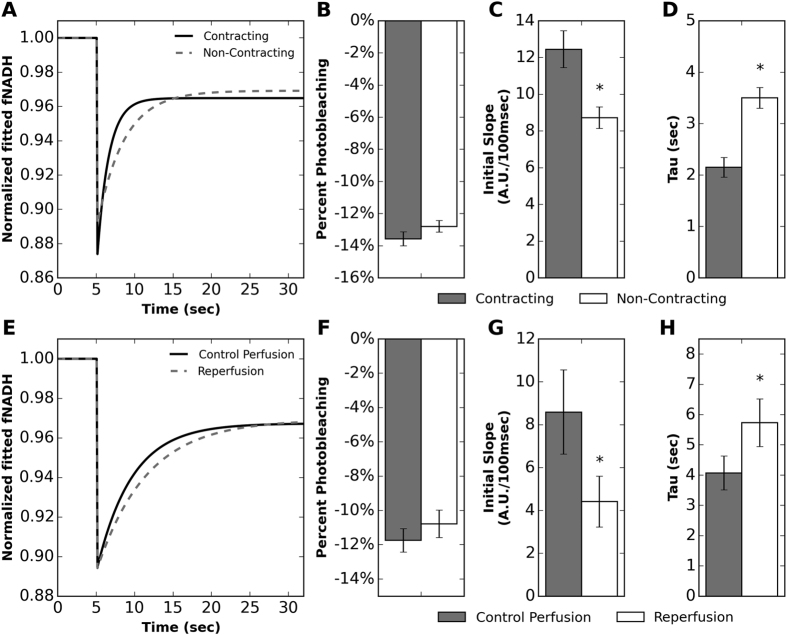
NADH ED-FRAP measurements (LP mode) showing the effect of contraction (top row) and ischemia/reperfusion (bottom row) on the rate of NADH production in perfused hearts. (**A**) Representative fNADH data were fitted (‘y’ in Equation 4) and plotted for NADH ED-FRAP applied to contracting hearts and non-contracting hearts electromechanically uncoupling with BDM. (**B**) Percent photobleaching was not significantly different between contracting and non-contracting hearts (p > 0.05, n = 6). (**C**) The initial slope of fNADH recovery was significantly higher in contracting hearts (p = 0.015, n = 6). (**D**) The time constant of fNADH recovery (tau) was significantly shorter in contracting hearts (p < 0.001, n = 6). (**E**) Representative fNADH data were fitted (‘y’ in Equation 4) and plotted for NADH ED-FRAP applied before ischemia and 10 min after reperfusion. Hearts were electromechanically uncoupled with BDM. (**F**) Percent photobleaching was not significantly different before ischemia and after reperfusion (p > 0.05, n = 5). (**G**) The initial slope of fNADH recovery was significantly lower after reperfusion (p = 0.04, n = 5). (**H**) The time constant of fNADH recovery (tau) was significantly longer after reperfusion (p = 0.049, n = 5).

The effect of actomyosin ATPase inhibition was studied using NADH ED-FRAP to determine if the rate of NADH production would correspond to a reduction in myocardial energy consumption resulting from diminished actomyosin ATPase activity. Typical fNADH recovery curves for contracting and non-contracting hearts after actomyosin ATPase inhibition with BDM are shown in Fig. 6A. A comparison of percent photobleaching between contracting hearts and noncontracting hearts (13.6 ± 0.43% vs 12.8 ± 0.36%) (Fig. 6B) did not reveal significant differences (p = 0.231). The initial slope of recovery was greater in hearts before inhibition (Fig. 6C) and tau was shorter (Fig. 6D), demonstrating significantly increased NADH and ATP production rates when contraction is not inhibited.

### Dehydrogenase activity and ischemia/reperfusion

Acute ischemia/reperfusion injury was studied using NADH ED-FRAP to determine if the rate of NADH production was impaired after reperfusion. Typical fNADH recovery curves before and after ischemia/reperfusion injury are shown in Fig. 6E. The initial rate of recovery was almost twice as fast before ischemia than after reperfusion (8.6 ± 2.0 vs 4.4 ± 1.2 A.U./100 msec, respectively) (p = 0.04) (Fig. 6G). The fNADH recovery time constant tau was also significantly longer (p = 0.049) after reperfusion than before ischemia (5.7 ± 0.8 vs 4.1 ± 0.6 sec, respectively) (Fig. 6H). Percent photobleaching was not significantly different (p = 0.419) before ischemia and after reperfusion (Fig. 6F).

## Discussion

We have shown that the recovery kinetics of fNADH after photobleaching reproducibly represent changes in NADH production during a variety of physiologic perturbations and provides insight into myocardial energy production beyond that of only monitoring changes in unbleached fNADH. In particular, unbleached fNADH represents the combination of NADH production and utilization but NADH ED-FRAP assesses the rate of NADH production, with the important assumption that NADH utilization does not change during the assessment. Indeed, a primary advantage of NADH ED-FRAP is that NADH production can be assessed without altering the physiological conditions of the tissue. We also identified optimal nondestructive NADH ED-FRAP parameters: 23.8 mJ of photobleaching light energy delivered with a pulse width of 6 msec and a duty cycle of 50%. These parameters provide reproducible assessments of dehydrogenase enzyme activity within epicardial tissue. Our studies also reveal that multiple applications of high total light energies should be avoided to prevent tissue photodamage.

NADH ED-FRAP parameters were optimized in studies that analyzed a range of delivered energies (TEDs) and four modes of NADH photobleaching. As presented in Fig. 2, percent photobleaching and the initial slope of fNADH recovery were dependent upon TED values below 18.2 mJ. Percent photobleaching and initial slope were not significantly different for TED values above 18.2 mJ. Similar results were reported by Combs and Balaban in isolated cardiac myocytes^2^. In those studies, the recovery rate of fNADH increased when the level of NADH photolysis increased by either changing the overall power of the laser or the number photobleaching pulses. These results emphasize that, for any set of experiments, a standard mode of photobleaching should be used for all measurements to reduce measurement variability. Our results also indicate that percent recovery and tau are likely not as sensitive to TED (Fig. 2C,D). While percent recovery was close to 100%, it is possible that the photobleaching of background, non-NADH fluorophores (~15% of NADH fluorescence) may have occurred. Similarly, any loss of the total NAD^+^: NADH pool could have prevented full fNADH recovery^3^. Although tau was dependent upon the photobleaching mode, the effect of TED on tau within each photobleaching mode was not significant (Fig. 2D). This suggests that tau could be a more robust indicator of dehydrogenase activity when systematic delivery of a specific TED cannot be guaranteed.

We observed that low power UV illumination (1.5 mW) used to image fNADH does not cause measurable photobleaching of epicardial tissue (Fig. 3A). This could be the result of an increase in NADH production to balance NADH photolysis or simply the result of negligible NADH photolysis by such low power light. We also observed that UV illumination for NADH photobleaching (500 mW and a TED of 23.8 mJ) applied using the long pulse (LP) mode is non-destructive and that several NADH ED-FRAP measurements can be obtained from the same site without considerably altering subsequent measurements (Figs 3B and 4C). After analyzing percent photobleaching, initial slope, tau, and percent recovery for all photobleaching modes, we chose the LP mode and 23.8 mJ for all subsequent experiments. With this mode, TED is situated within the percent photobleaching plateau (Fig. 2A) and the pulse width of 6 msec is compatible with most software and hardware for the development of custom NADH ED-FRAP applications. Shorter pulse widths, such as 200 μsec for the SP mode, often require specialized hardware and software.

It is generally understood that enzyme activity is positively correlated with temperature, with dependencies ranging from sub-freezing to high temperatures^25^,^26^,^27^. This correlation is the basis for therapeutic hypothermia in patients suffering from cardiac arrest and the lowering of core temperature during cardiac surgeries that require cardiopulmonary bypass^28^,^29^. When myocardial temperature is lowered, heart rate, contractile force, oxygen consumption, and, ultimately, ATP utilization all drop dramatically. A reduction in myocardial temperature decreases both the steady state utilization and production rate of ATP, as well as decreases the upstream utilization and production of NADH. We therefore studied hearts perfused at three different temperatures (~22, ~30 and ~37 °C) to test whether or not NADH ED-FRAP would detect a temperature-dependent slowing of NADH production. Indeed, the recovery kinetics of fNADH dropped as temperature dropped, indicating a significant decrease in NADH production rate (Fig. 5). Overall, the relationship between initial slope and temperature (Fig. 5A) and tau and temperature (Fig. 5B) was proportional, although tau appeared to increase more between 30 & 22 °C than between 37 & 30 °C, indicating that the relationship may have an exponential component. Additionally, GDH activity was measured as an independent validation of the effect of temperature on a mitochondrial enzyme actively involved in NADH production. The progressive drop in GDH activity as temperature was lowered, which we measured using a standard molecular assay, indicates that fNADH recovery kinetics (initial slope and tau) during NADH ED-FRAP mirrors changes (Fig. 5D) in the activity of a NADH producing enzyme found in the mitochondria.

Total myocardial energy consumption is determined by actomyosin crossbridge cycling (~76%), calcium transport (~15%), and the maintenance of sarcolemmal potential by the Na^+^/K^+^ ATPase (~9%)^30^,^31^. Thus, actomyosin ATPase inhibition via electromechanical uncoupling significantly diminishes myocardial energy consumption, which slows ATP production and slows upstream NADH production. We have previously shown that fNADH rises rapidly in contracting hearts after the termination of flow to the aorta, reaching a plateau (full reduction of NADH) within ~90 sec. In contrast, NADH accumulation during ischemia in electromechanically uncoupled hearts is much slower and reaches a plateau after 5–10 min^13^,^32^. These differences in the rate of NADH production were confirmed by the higher initial slopes and shorter values of tau that we measured via NADH ED-FRAP (Fig. 6C,D). Although the motion of contraction introduced some oscillation in fluorescence acquired during NADH ED-FRAP, the oscillation frequency was much higher than the slower average rise of fNADH, providing for adequate analysis of recovery kinetics.

Our results indicate that NADH ED-FRAP is a useful approach for measuring NADH production after metabolic insults and provide additional insight into the sustained effects of ischemia/reperfusion injury in isolated perfused hearts. Ischemia/reperfusion injury is a complex phenomenon that begins when mitochondrial oxygen availability is compromised during ischemia. Mitochondrial NADH subsequently rises^13^,^33^ and fatty acid and carbohydrate oxidation are halted^34^. After reperfusion, cardiac energy production remains compromised, likely due to an imbalance between glycolysis and full glucose oxidation^34^,^35^. Mitochondrial damage caused by reperfusion is another important mechanism of impaired myocardial energetics, which is thought to be caused by intracellular Ca^2+^ overload^36^, mitochondrial ROS production, and opening the mitochondrial permeability transition pore^37^,^38^,^39^. Mitochondrial damage would likely motivate dysfunction of complex I, III and IV of the electron transport chain (ETC), which has been shown to persist for at least 10–15 min after reperfusion. Such dysfunction has been ascribed to a greater disintegration and loss of cardiolipin^40^,^41^,^42^,^43^, a key phospholipid located in the mitochondrial membrane and is thought to serve as a proton trap within the ETC^44^,^45^. ROS-induced oxidative damage during metabolic insults can also damage cardiolipin^41^,^42^, which may further contribute to mitochondrial dysfunction after reperfusion.

Overall, our results are consistent with the known outcomes of reperfusion injury, where we observed a significant difference before and after reperfusion in both the initial slope of fNADH recovery (Fig. 6G) and the recovery time constant tau (Fig. 6H). These data provide the interesting observation that, even after 10 min of reperfusion, the rate of NADH production remained lower than the pre-ischemic level, likely the result of sustained mitochondrial damage. It is interesting that low levels of NADH production were maintained even though heart rate returned to the pre-ischemic level: 196 ± 12 bpm before ischemia and 183 ± 12 bpm after ischemia (p = 0.096). It is unlikely that the imposed 20 min of global ischemia confounded (other than the injury itself) the NADH ED-FRAP signal observed after 10 min of reperfusion given the rapid oxidation of NADH and restoration of fNADH to a level similar to that before ischemia, analogous to previous studies of ischemia in isolated hearts^46^,^47^,^48^.

## Conclusions

Ultraviolet energy delivered between 21–23.8 mJ provided consistent NADH photobleaching within epicardial tissue for the assessment of NADH production rate during multiple metabolic perturbations. As perfusate temperature was reduced the kinetics of fNADH recovery after photobleaching directly correlated with glutamate dehydrogenase activity, with longer recovery of fNADH corresponding to lower glutamate dehydrogenase activity. fNADH recovery kinetics after photobleaching remained significantly impaired after 10 min of reperfusion after global ischemia, indicating sustained impact of ischemia/reperfusion on dehydrogenase activity. High rates of NADH production were associated with physiologic temperature and the greater energetic demands of contraction. Overall, our results indicate that myocardial NADH ED-FRAP is a useful non-destructive approach for assessing dehydrogenase activity within living myocardium.

## Limitations

Potential limitations include the risk of ischemic damage during the heart excision and cannulation, thus the time from excision to cannulation was minimized. Most excised heart preparations are prone to oxygen limitations, in part because the perfusate does not contain red blood cells^31^. However, red blood cells are typically not added to perfusate because of the dominating optical absorption of haemoglobin (250–650 nm range). Another potential limitation is that fNADH was imaged from a thin layer of epicardial tissue and may not reflect transmural gradients of dehydrogenase activity, which is a relevant concern. This could be especially important during the first moments of ischemia because ischemia begins within endocardial tissue and progresses as an anoxic wave to the epicardium^49^. The rate and spatial extent of the progression of this wave is variable and modulated by factors such as wall stress and myocardial perfusion^50^,^51^,^52^. However, in our studies NADH recovery kinetics were measured after 10 min of reperfusion, an interval after which transmural gradients were likely to have stabilized. Finally, our fNADH measurements do not discriminate between NADH fluorescence and NADPH fluorescence. Nonetheless, the contribution of NADPH fluorescence in such measurements is thought to be insignificant due to its much lower concentration compared to NADH^53^,^54^ and minimal fluorescence enhancement within the mitochondria^55^.

## Materials and Methods

### Perfused heart preparations

Animal protocols were approved by The George Washington University’s Animal Care and Use Committee. All experiments were performed in accordance with the Guide for the Care and Use of Laboratory Animals published by the National Institutes of Health. Sprague-Dawley rats (315 ± 8.8 g, either sex) were anesthetized via an intraperitoneal injection of Telazol (40 mg/kg). Upon the cessation of pain reflexes, hearts were quickly excised, cannulated via the aorta, and Langendorff perfused at constant pressure (70 mmHg) and temperature (37 °C, except as noted) with an oxygenated (95% O_2_, 5% CO_2_) Krebs-Henseleit solution, containing, in mM: 118 NaCl, 4.7 KCl, 1.25 CaCl_2_, 0.57 MgSO_4_, 1.17 KH_2_PO_4_, 25 NaHCO_3_, 6 glucose and 500 mU/L insulin, pH = 7.4. Exogenous fatty acids were not added to the perfusate mainly because in isolated heart preparations the availability of endogenous fat is sufficient to properly maintain cardiac mechanical function for at least 60 min^56^, which provided for the completion of our NADH ED-FRAP protocols. For most experiments, the actomyosin ATPase inhibitor 2,3-butanedione monoxime (BDM, 15 mM) was administered to electromechanically uncouple the hearts to minimize motion artifacts during fluorescence imaging^57^,^58^. An electrocardiogram (ECG) was continuously acquired using a bioamplifier (Dagan EX4-400) and a PowerLab data acquisition system (AD Instruments).

### NADH ED-FRAP illumination and imaging

Two UV LED spotlights (PLS-0365-030-S & LCS-0365-11-22, Mightex Systems) provided light (367 ± 5.5 nm) to illuminate the epicardium. A low power spotlight (1.5 mW) was used for continuous NADH fluorescence (fNADH) imaging and a high power spotlight (500 mW) was used to photobleach epicardial NADH. The high power spotlight illuminated epicardial regions of approximately 7 mm^2^ (Fig. 1A). Prior to each experiment, a single image was acquired with the high power spotlight at low power (0.71 mW) to locate epicardial regions for subsequent fNADH recovery analysis. Emitted epicardial fluorescence was band-pass filtered (475 ± 25 nm, peak 460 nm^18^) and imaged at 10 Hz using a CCD camera (Andor iXon DV860). A background image (no lights) and a reference image (low power light source on, but no heart) were acquired before beginning each study. These images were stored and used for off-line analysis to automatically remove the baseline counts for each pixel. The lights and camera were synchronized using a custom LabVIEW (National Instruments) program. A typical NADH ED-FRAP protocol was: 5 sec of baseline (control) imaging, a brief period of NADH photobleaching (0.30–8.0 sec, described below), and 95 sec of continuous imaging to record fluorescence recovery (Fig. 1B). Percent photobleaching was calculated with respect to the average fNADH level during the 5 sec of baseline imaging before photobleaching. One-dimensional spatial fNADH profiles were computed by interpolating fNADH along a user-defined 3.3mm line (one pixel wide) that passed through a photobleached area (Fig. 1C).

### Full range of fNADH: fully oxidized to fully reduced

The range of fluorescence from fully oxidized to fully reduced NADH was measured to determine the percentage of the total NADH pool that was photobleached. Baseline fNADH was imaged and then perfusate flow to the aorta was terminated, causing global ischemia and full reduction of the mitochondrial NADH pool (Fig. 1B). fNADH was acquired until fluorescence plateaued (97 ± 8 sec after initiating global ischemia). In other hearts, after imaging baseline (control) fNADH, carbonyl cyanide m-chlorophenylhydrazone (CCCP, 10 μM) was added to the perfusate to dissipate the mitochondrial proton gradient, fully oxidizing NADH, causing the fNADH signal to drop^59^. The NADH pool was considered to be fully oxidized when both the fNADH signal plateaued to a minimum (204 ± 8 sec after fNADH began to fall) and illumination with the high power UV LED did not cause an additional drop in fNADH. After acquiring the fNADH maxima and minima from multiple studies the data were normalized using the following equation:


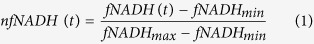


where fNADH(t) is the fNADH signal acquired during a study, fNADH_max_ is the maximum fluorescence intensity when NADH is fully reduced and fNADH_min_ is the minimum fluorescence intensity when NADH is fully oxidized.

### Optimization of NADH ED-FRAP parameters

Multiple variables were studied to determine optimal photobleaching parameters. The total energy delivered (TED) for photobleaching was optimized by increasing TED from 2.8 to 28 mJ while analyzing 4 key parameters (Fig. 1D): (1) Percent photobleaching, the drop in fNADH that occurs immediately following illumination with high-intensity UV light; (2) Tau, the time constant of the rise (τ) of fNADH after photobleaching; (3) Initial slope, the recovery rate of fNADH during the first second after photobleaching; and (4) Percent recovery, the degree to which fNADH recovered to baseline.

Approaches for delivering optimal TED for photobleaching were defined to study how TED might best be applied. As such, four photobleaching modes were defined with specific light power, duty cycle, and pulse widths (Table 1), with each providing equal TED. For example, while maintaining TED at 23.8 mJ, the effect of decreasing the length of each individual light pulse was tested by decreasing the pulse width from 6 msec (Long Pulse, LP) to 200 μsec (Short Pulse, SP), while light power (500 mW), duty cycle (50%), and total bleaching time (5.1 sec) remained constant between the two conditions (Table 1). Next, 375 mW (Low Light Power, LLP) was used to measure the effect of reducing the light power by 25%. Duty cycle remained at 50% with a pulse width of 6 msec. This increased total photobleaching time to 6.8 sec while maintaining TED at 23.8 mJ (Table 1). Finally, the effect of a single pulse (1P) for photobleaching was tested, which dropped total bleaching time to 2.55 sec while maintaining a TED of 23.8 mJ. Each photobleaching mode (LP, SP, LLP, and 1 P) was tested at each TED (2.8 to 28 mJ). The number of pulses, duty cycle, and pulse width for each photobleaching mode was controlled using our LabVIEW program, which also synchronized with the camera and ensured that the camera did not acquire images during photobleaching to prevent damage to the CCD.

### Measurement of tissue viability and cellular damage

The effect of UV illumination on tissue viability and cellular morphology was assessed using triphenyltetrazolium chloride (TTC) tissue staining and haematoxylin and eosin (H&E) histology. Epicardial sites where NADH ED-FRAP was applied using one of the four modes of photobleaching (Table 1) were marked with permanent blue ink. As a positive control, several hearts were also subjected to 10 min of continuous UV illumination at the maximum LED power (500 mW). Heart perfusion was maintained for at least two hours after completing the UV illumination protocols to provide time for the rundown of cellular metabolism and any cellular necrosis to occur that might have resulted from UV photodamage. Hearts were then incubated in a TTC solution (42 mM) at 37 °C for 10 min to reveal non-viable tissue. TTC stains metabolically active tissue a deep red color, with metabolically inactive or damaged tissue presenting as a pale tan color^60^. Tissue damage was assessed in this way for three hearts subjected to each photobleaching mode. A subset of hearts was fixed in 10% formalin and cross-section slices were prepared for H&E staining to examine cell morphology. Haematoxylin stains the nuclei of cells as blue/purple and eosin stains nonspecifically stains proteins as pink^61^.

### Dehydrogenase activity and temperature

Low temperatures slow the rate of enzyme-catalyzed reactions so we tested whether NADH ED-FRAP would reveal the effect of a drop in perfusate temperature on the rate of NADH production in perfused hearts. These experiments were performed using the LP mode of photobleaching with a TED of 23.8 mJ (Table 1). Perfusate temperature was set at either 22 ± 0.18, or 30 ± 0.16, or 36.6 ± 0.11 °C for each study. The rate of fNADH recovery was measured after multiple rounds of photobleaching at each perfusate temperature.

Glutamate dehydrogenase (GDH) activity was determined using enriched mitochondrial fractions to correlate the rate of fNADH recovery measured after photobleaching with the activity of an NADH producing enzyme that resides within the mitochondria. Enriched mitochondrial fractions were extracted from ventricular tissue and 0.08–0.13 μg of protein was added to a cuvette containing, in mM: 50 TEA, 2.5 EDTA, 100 ammonium acetate, 1 ADP, 0.2 NADH, and 2 kU/L lactate dehydrogenase, in a final volume of 1 mL, pH = 7.6. Background absorbance was measured at 340 nm for 1 min in a spectrophotometer (SpectraMax Plus 384, Molecular Devices). The addition of 2-oxogluterate (7 mM) initiated substrate-dependent activity and A_340_ was measured at 340 nm for 2 min. GDH activity was calculated using an NADH millimolar extinction coefficient of 6.23. Average temperatures for GDH activity measurements were 22 ± 0.01, 30 ± 0.01, and 37 ± 0.01 °C.

### Dehydrogenase activity and contraction

The actomyosin ATPase is a major consumer of myocyte ATP and its rate of ATP hydrolysis modulates the rate of mitochondrial NADH production. In a separate set of studies the impact of actomyosin ATPase activity on fNADH recovery after photobleaching was measured. Before administering the actomyosin ATPase inhibitor BDM, NADH ED-FRAP was applied to contracting perfused hearts. BDM was then administered and hearts were monitored until the cessation of contractions and a stable heart rate were observed. NADH ED-FRAP was performed again to compare fNADH recovery kinetics before and after actomyosin ATPase inhibition.

### Dehydrogenase activity and ischemia/reperfusion

Acute ischemia/reperfusion injury has been reported to diminish mitochondrial ATP production^62^,^63^. If so, then the rate of NADH production may also be lower after ischemia/reperfusion injury. We tested this hypothesis in a separate set of perfused heart studies using NADH ED-FRAP. Baseline fNADH recovery kinetics were measured before aortic flow was halted for 20 min. Hearts were reperfused for 10 min, after which fNADH recovery kinetics were measured again via NADH ED-FRAP. fNADH recovery kinetics measured before and after global ischemia/reperfusion were then compared.

### Data analysis

NADH fluorescence images were recorded using Solis imaging software (Andor) and extracted using ImageJ. To compute an fNADH signal, a region of interest (ROI) within the photobleached region was selected and all pixels within that ROI were averaged to provide a temporal fNADH signal: fNADH(t). Percent photobleaching and percent recovery with respect to baseline were calculated using the following equations:


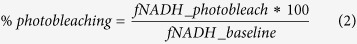



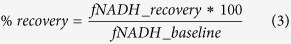


where fNADH_photobleach is the minimum fluorescence intensity after high power UV light was applied to the epicardial surface, fNADH_recovery is the fluorescence intensity when the recovery reached a steady-state, and fNADH_baseline is the average fluorescence intensity during the first 5 sec before photobleaching.

The time constant of fNADH recovery after photobleaching (tau) was computed using a least-squares fit of the following equation to the fluorescence ROI data (fNADH(t)) acquired during the fNADH recovery phase (Fig. 1B):


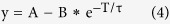


where A is the baseline value from which the signal is rising, B is the signal amplitude, T is time (sec), and tau (τ, 1/sec) is the inverse of the exponential rate constant of rise, or the time constant, when the amplitude reaches 63% of recovery. The initial slope of the fNADH recovery curve was also calculated by computing the first derivative of the fitted recovery line (y, Equation 4) averaged over the first second of recovery. Data were analyzed using custom Matlab scripts and plotted using Spyder (Python 2.7).

### Statistical analysis

Statistical analyses were performed using Minitab 16 and Microsoft Excel. Data are presented as mean ± standard error of the mean. A paired t-test or an analysis of variance (ANOVA) general linear model (GLM) with Tukey post-hoc test, as appropriate, was used to compare between different groups. Significance was defined as p < 0.05. An Anderson–Darling test was performed to determine normality in the data.

## Additional Information

**How to cite this article:** Moreno, A. *et al*. Enzyme-dependent fluorescence recovery of NADH after photobleaching to assess dehydrogenase activity of isolated perfused hearts. *Sci. Rep.* **7**, 45744; doi: 10.1038/srep45744 (2017).

**Publisher's note:** Springer Nature remains neutral with regard to jurisdictional claims in published maps and institutional affiliations.

## Figures and Tables

**Table 1:** Parameters for the four photobleaching modes used during the photobleaching optimization studies (Fig. 2).

	Light Power	Duty Cycle	Pulse Width	Energy Per Pulse	Total Bleaching Time	# of Pulses	Total Energy Delivered
Long Pulse (LP)	500 mW	50%	6 msec	28 μJ	5.1 sec	850	23.8 mJ
Short Pulse (SP)	500 mW	50%	200 μsec	0.934 μJ	5.1 sec	25482	23.8 mJ
Low Light Power (LLP)	375 mW	50%	6 msec	21 μJ	6.8 sec	1132	23.8 mJ
Single Pulse (1P)	500 mW	100%	1 pulse	Continuous	2.55 sec	1	23.8 mJ

Results indicated that the LP mode was the optimal photobleaching mode for NADH ED-FRAP.
